# The positive effect of short-term nano-curcumin therapy on insulin resistance and serum levels of afamin in patients with metabolic syndrome

**Published:** 2021

**Authors:** Nejat Kheiripour, Zahra Khodamoradi, Akram Ranjbar, Shiva Borzouei

**Affiliations:** 1 *Research Center for Biochemistry and Nutrition in Metabolic Diseases, Kashan University of Medical Sciences, Kashan, Iran*; 2 *Student Research Committee, Hamadan University of Medical Sciences, Hamadan, * *‎* *Iran*; 3 *Toxicology and Pharmacology Department, School of Pharmacy, Hamadan University of Medical Sciences, Hamadan, Iran*; 4 *Clinical Research Development Unit of Shahid Beheshti Hospital, Hamadan University of Medical science, Hamadan, Iran*

**Keywords:** Nano-curcumin, Metabolic syndrome, Insulin resistance, Afamin

## Abstract

**Objective:**

Metabolic syndrome (MS) is a cluster of cardio-metabolic risk factors. MS is known as a highly prevalent disease worldwide. According to the existing evidence, consuming curcumin has positive effects on lipids profile, glucose, and body weight. This study aimed to evaluate the effects of nano-curcumin therapy on insulin resistance and serum level of afamin in patients with MS.

**Materials and Methods:**

Thirty MS patients (15 males and 15 females) received 80 mg/daily nano-curcumin for two months. The samples of fasting blood were collected from the participants at the beginning and 60 days after initiation of the intervention to measure biomarkers**.**

**Results:**

Comparing pre- and post-treatment with nano-curcumin values revealed a significant decrease in fasting plasma glucose (FPG) (p=0.017), insulin, homeostatic model assessment of insulin resistance (HOMA-IR) (p=0.006), and afamin (p=0.047). Moreover, there was a significantly negative relationship between afamin and high-density lipoprotein cholesterol (HDL-C) (p=0.044), as well as a significantly positive relationship between afamin and systolic (SBP) (p<0.001) and diastolic (DBP) (p<0.001) blood pressures.

**Conclusion:**

Results suggest that taking nano-curcumin for 60 days may have positive effects on afamin, FPG, insulin, and HOMA-IR in patients with MS, but would not significantly affect other metabolic profiles. More studies with larger sample sizes are required to confirm these findings.

## Introduction

Metabolic syndrome (MS) is a complex metabolic disorder that includes a combination of clinical symptoms: hyperglycemia, high triglyceride levels, low HDL cholesterol, elevated blood pressure, and central obesity (Javandoost et al., 2018 ▶; Alberti et al., 2009 ▶). Insulin resistance (IR) refers to insensitivity to insulin-mediated glucose disposal. IR is a major component of MS (Hosseinpanah et al., 2012 ▶). IR and MS prevalence is increasing, especially in developing countries with a prevalence estimate range of 20 to 40% depending on the population (Watson et al., 2019 ▶; Almobarak et al., 2020 ▶). Both IR and MS are powerful risk factors for the development of cardiovascular disease and type 2 diabetes (Ko et al., 2019 ▶).

 Discovered in 1994, afamin is a glycoprotein which is composed of human serum albumin, alpha-photoprotein, and vitamin D-binding protein. Afamin is found on chromosome 4q11-q13 in humans. The molecular weight of afamin is 87000 Daltons, with 15% carbohydrate content and 55% amino acid sequence similarity to albumin (Lichenstein et al., 1994 ▶). 

Afamin is a glycoprotein that binds vitamin E (α-tocopherol) in human plasma and it is secreted mainly from the liver, kidneys, testicles, and ovaries. It has been shown that blood afamin levels are related to many diseases such as obesity, pregnancy complications, and polycystic ovary syndrome, type-2 diabetes, hypertension, and dyslipidemia. Also, afamin has been known to be a tumor marker for some cancer types including ovarian cancer where afamin is likely involved in regulation of the bone metabolism and signaling pathways (Dieplinger & Dieplinger, 2015 ▶). Previous studies showed the relationship between the prevalence of MS and afamin concentrations (Kollerits et al., 2017 ▶). An association between increased afamin concentration and IR was observed in patients with the polycystic ovarian syndrome (Seeber et al., 2014 ▶). 

Natural plant products have been used throughout human history for various purposes. Curcumin [1, 7-bis (4-hydroxy-3-methoxyphenyl)-1, 6-heptadiene-3, 5-Dione] (Cur) is a highly active component of turmeric root (Ghasemi, et al., 2019 ▶). This yellow curry spice has long been used as traditional medicine (Sharangi and Acharya, 2018 ▶). Cur, as a herbal medicine, has no major side effects and it is regarded as a beneficial therapeutic agent in traditional medicine. 

According to the results of some recent studies, Cur - encapsulated nano-systems likely increase Cur stability and bioavailability, and prevent its physical degradation and absorption in the gastrointestinal tract (Hatamipour et al., 2019 ▶; Ganugula et al., 2017 ▶). 

Changes in HOMA-IR, abnormal concentrations of insulin and lipid profiles, as well as changes in the level of plasma afamin, are important features in MS; however, few studies were carried out on the relationships between nano-curcumin administration and the above-mentioned factors. This study was conducted to investigate the effect of nano-curcumin on changes in IR and serum levels of afamin in patients with MS.

## Materials and Methods

**Study design and population**

The patient population initially consisted of 44 participants who were referred for outpatient care in the Shahid Beheshti hospital of Hamadan, Iran; 14 people were excluded from the study due to lack of metabolic syndrome criteria. Thyroid disease, significant autoimmune disease, malignant disease, chronic kidney disease (CKD), cirrhosis, AIDS, receiving glucocorticoid treatment, pregnancy/lactation, and receiving insulin.

The remaining 30 participants entered the study (15 men and 15 women, aged 41.768.02 years). Participants underwent a standard evaluation, which included medical history, physical examination, and anthropometric measurement performed before and at the end of the study on participants receiving nano-curcumin capsules (Exir Nano Sina Company, Iran. IRC: 1228225765) (80 mg/day) for eight weeks.

**Biochemical measures**

Blood samples were drawn after a 12-hour overnight fast, for biomarkers measurement at the beginning and the end of the intervention. 

The enzymatic colorimetric method was adopted to measure fasting plasma glucose (FPG) using glucose oxidase. 

Commercial Enzymatic reagents were used to measure serum total cholesterol and triglyceride (TG) concentrations (Pars Azmoon, Iran).

High-density lipoprotein cholesterol (HDL-C) was measured after precipitation of the *Apo*lipoproteins and B-containing lipoproteins with phosphotungstic acid. Low-density lipoprotein cholesterol was calculated from serum total cholesterol, TG and HDL-C, except when TG concentration was 400 mg/dl and above.

Ultrasensitive Enzyme-linked radioimmunoassay method was used to measure the concentration of serum insulin (Mercodia, Uppsala, Sweden).

The concentrations of serum afamin were determined using a commercial ELISA kit (My Biosource, San Diego, United States).

**Definition**

Joint Interim Statement (JIS) defines MS as the presence of at least three out of the following five risk factors (Alberti et al., 2009 ▶):

1. Abdominal obesity with a waist circumference ≥95 cm for both genders according to population and country-specific cut off points for the Iranians (Azizi et al., 2010 ▶). 

1. FPG≥100 mg/dl or drug treatment. 

2. Fasting TG≥150 mg/dl or drug treatment.

3. Fasting HDL –C<50 mg/dl in women and HDL–C<40 mg/dl in men.

4. SBP≥130 mmHg, or DBP≥85 mmHg which indicates hypertension or antihypertensive drug treatment.

Insulin resistance is calculated using the HOMA model as a standard reference according to the following formula (Wallace, Levy, & Matthews, 2004 ▶): 

HOMA-IR = [(fasting insulin level (mU/l))× (FPG (mmol/l))]/22.5 

**Ethical issues**

The research followed the tenets of the Declaration of Helsinki. The Ethics Committee of Hamadan University of Medical Sciences approved this study (IR.UMSHA.REC.1397.873). All study protocols were approved by Hamadan University of Medical Sciences and registered as a clinical trial at the Iranian Registry of Clinical Trials (IRCTID: IRCT20120215009014N277; http://irct.ir/trial/39176; registration date 2019-05-13); also, written informed consent was obtained from all participants before any intervention.

**Statistical analysis**

Data analysis was performed using SPSS version 22.0 (SPSS Inc., Chicago-USA) and GraphPad Prism version 6.0 (GraphPad Software, San Diego-USA). To ensure a normal distribution of the variables, the Kolmogorov-Smirnov test was used. The results are expressed as number, percent, and mean±standard deviation. Student’s t-test (two-tailed and paired) and correlation analysis (Pearson) were run to analyze the data. A p value of <0.05 was considered significant.

## Results

The study participants were 15 men and 15 women with a mean age of 41.76±8.02 years. No significant difference was observed in the body mass index (p=0.816), waist circumference (p=0.612), SBP (p=0.341) and DBP (p=0.121) at the beginning and eight weeks after the treatment (Table 1). 

**Changes in lipid profile in serum**

There were some changes in HDL-C, LDL-C, TC, and TG after treatment with nono-curcumin; however, the differences were not statistically significant (Table 1). 

**Changes in FPG, insulin, HOMA-IR, and afamin**

**Figure 1:**
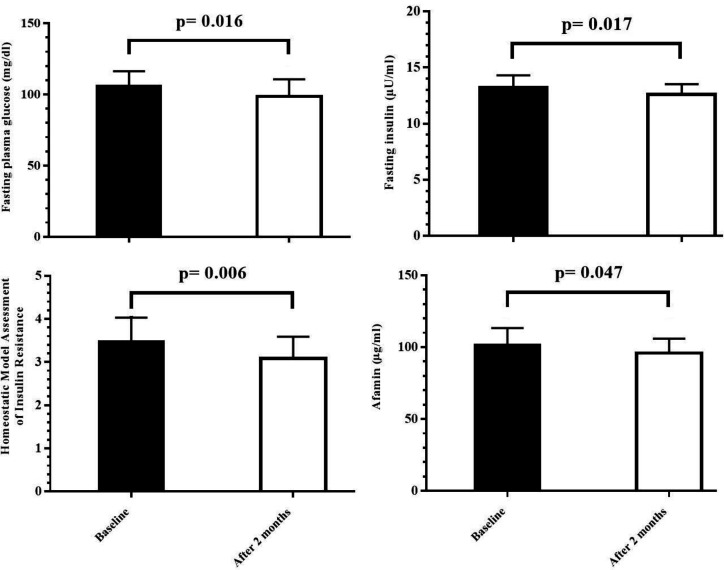
Levels of fasting plasma glucose, insulin, HOMA-IR, and afamin at baseline and after two months of nano-curcumin therapy in metabolic syndrome patients. The results are expressed as mean±SD. p-values<0.05 were considered statistically significant

The results showed a significant reduction in FPG (p=0.016) and HOMA-IR (p=0.006) after nano-curcumin treatment. Also, the difference between insulin levels before and after the intervention was also statistically significant (p=0.017) (Figure 1). 

We also investigated the effects of nano-curcumin on the afamin in the serum of MS patients. We observed significant differences between pre- and post-treatment levels of afamin (p=0.047) (Figure 1).

**Comparison of the factors studied in** **males** **and females**

**Figure 2:**
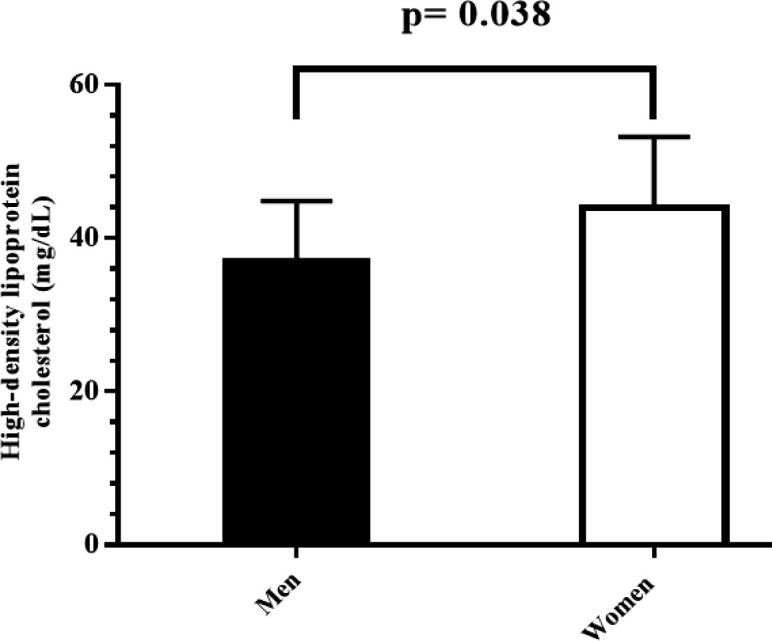
Comparison of the level of HDL after two months of nanourcumin therapy between men and women. The results expressed as mean±SD. p-values <0.05 were considered statistically significant

It is worth noting that no significant difference was observed in any of the factors in men and women. The exception was HDL, which showed a significant difference following the eight-week treatment period in male and female patients (Figure 2).

**Table 1:** Health and demographic data of MS subjects and the effects of a single daily dose (80 mg) of nano-curcumin for two months on the status of MS-related indices (n=30)

**p-value**	**After two months**	**Baseline**	**Variables**
0.926	91.48±14.67	91.83±14.41	Weight (kg)
1.000	168.60±10.30	168.60±10.30	Height (cm)
0.612	108.30±7.41	109.30±7.77	Waist circumference (cm)
0.816	32.07±4.15	32.33±4.23	BMI (kg/m^2^)
0.341	115.50±10.03	118.33±12.68	SBP (mmHg)
0.121	78.66±8.19	81.83±7.36	DBP (mmHg)
0.786	190.06±32.89	188.37±30.54	TC (mg/dl)
0.150	40.52±9.08	36.83±10.44	HDL-C (mg/dl)
0.459	108.23±20.46	112.51±23.78	LDL-C (mg/dl)
0.320	176.30±11.34	196.97±17.15	TG (mg/dl)

The results expressed as mean±SD. SD: Standard deviation; MS: Metabolic Syndrome; BMI: Body mass index; SBP: Systolic blood pressure; DBP: Diastolic blood pressure; TC: total cholesterol; HDL-C: High-density lipoprotein cholesterol; LDL-C: Low-density lipoprotein cholesterol; TG: Triglyceride.

**Pearson correlation analysis between afamin and metabolic syndrome-related indices (after** **eight weeks** **of nano-curcumin therapy)**

**Figure 3:**
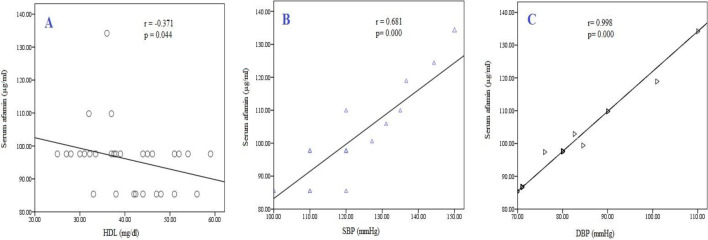
The correlation between serum afamin and (A) high density lipoprotein cholesterol (HDL-C) (r=-0.371; p=0.044), (B) Systolic blood pressure (SBP) (r=0.681; p<0.001), and (C) Diastolic blood pressure (DBP) (r=0.998; p<0.001) after two months of nano-curcumin therapy in metabolic syndrome patients

After eight weeks of nano-curcumin therapy, a significantly negative correlation existed between serum afamin concentrations and HDL-C (p=0.044), and there was a positive correlation between afamin with SBP and DBP (p<0.001) (Figure 3). There was a non-significant negative relationship between afamin concentrations and insulin, TC and LDL, and a non-significant positive relationship between afamin and FPG, HOMA-IR, TG, and waist circumference (cm) (Table 2).

**Table 2:** Pearson correlation analysis between afamin and metabolic syndrome-related indices (after two months of nano-curcumin therapy) (n=30)

**Factors **	**The correlation coefficient**	**p-value**
BMI (kg/m^2^)	0.296	0.112
Waist circumference (cm)	0.180	0.342
SBP (mmHg)	0.681	<0.001
DBP (mmHg)	0.998	<0.001
FPG (mg/dl)	0.075	0.694
Insulin (µU/ml)	-0.135	0.477
HOMA-IR	0.013	0.981
TG (mg/dl)	0.005	0.981
TC (mg/dl)	-0.151	0.427
HDL (mg/dl)	-0.371	0.044*
LDL (mg/dl)	-0.121	0.523

*indicates significance at p<0.05.

## Discussion

According to previous studies, Cur has antidiabetic, anti-oxidant, anti-inflammation, and anti-cancer effects (Xu et al., 2018 ▶). Thus, the purpose of this study was to investigate the effects of nano-curcumin therapy on FPG and lipid profile, insulin, HOMA-IR, afamin hormone, and MS indicators.

This study showed that eight weeks of nano-curcumin consumption by the patients with MS had useful effects on insulin, improved FPG and HOMA-IR, and attenuated afamin. 

Cur has a very low stability and bioavailability. Since it cannot be easily dissolved in water, it is quickly metabolized, poorly absorbed in the intestine, and because of systemic elimination, its level remains low in the plasma. New studies revealed the nano-encapsulated curcuminoids increased absorption rate in the gastrointestinal tract stability by the way those prolonge circulation half-life and increase bioavailability. Also, it was revealed that the water solubility of nano-curcumin is considerably higher compared to previous forms. Also, it was reported that within less than 15 min after oral administration of nanocurcumin, the capsulated nanocurcumin opens in the stomach, and is diffused to the small intestine, which makes this product more bioavailable than its counterparts (Rahimi et al., 2014 ▶; Hatamipour et al., 2019 ▶; Du et al., 2013 ▶). According to the results of this study, it can be suggested that nano-curcumin can be a favorable therapeutic agent against MS.

Insulin is an anabolic hormone which is generated by pancreatic beta cells. Proper tissue development, growth, and glucose homeostasis are highly dependent on insulin. The absorption of glucose by muscle and fat cells, liver glycogen synthesis, protein synthesis, lipogenesis, and lipoprotein lipase activity are increased by insulin. Recent studies reported that most patients with MS have IR. It is widely believed that IR and hyperinsulinemia can be considered major causes of MS (Cornier et al., 2008 ▶). In the present study, we found that nano-curcumin therapy for two months can reduce insulin concentration in MS patients. Consistent with the results of this study, Nishiyama et al. (Nishiyama et al., 2005 ▶) indicated that Cur induces the activity of PPAR-γ. It was reported that PPAR-γ can be mentioned as a necessary mediator to keep the sensitivity to the whole body’s insulin. Activation of PPAR-γ acutely improved insulin and its sensitivity and thus resulted in decreased FPG and HOMA-IR (Kintscher & Law, 2005 ▶).

The results of other studies showed that nano-curcumin has antidiabetic effects and reduces FPG (Rahimi et al., 2016 ▶). In the same vein, in the present clinical trial, we found that nano-curcumin therapy decreases FPG after two months. 

It was reported that increased levels of the serum afamin were related to IR and MS (Seeber, et al., 2014 ▶). The results of the present study indicated that afamin levels are very high in serum of MS patients which is in line with those of other studies that reported a relationship between the increased levels of afamin and MS in young women which are likely to be regarded as an independent anticipator for the progression of MS (Seeber, et al., 2014 ▶). Also, this study showed that following 60-day treatment with nano-curcumin, a significant difference in serum levels of afamin was found between pretreatment and post-treatment values. 

Elevated SBP and DBP levels are well-established risk factors for MS (Kjeldsen, etal., 2008 ▶). Moreover, evaluating the relationship between serum afamin and some MS features showed significant positive correlations between increased afamin levels and severity of MS indicators such as SBP and DBP; while inverse significant correlations were detected between afamin concentrations and HDL-C. Our findings are in line with a previous study showing that afamin levels correlated with the total number of metabolic syndrome criteria (Kronenberg et al., 2014 ▶). 

Therefore, this study showed that the positive effects of nano-curcumin in MS and its complications such as IR and increased FPG might be associated with the reduction of afamin levels in the serum of MS patients. This study had some limitations, including small sample sizes; also, we did not assess plasma inflammatory factors and oxidative stress levels.

Taken together, treatment with nano-curcumin led to reduction of FPG, improvement of sensitivity to insulin, and reduction in HOMA-IR levels in MS patients. Furthermore, the results of this study indicated that nano-curcumin administration could significantly decrease the level of afamin in the serum of MS patients. Thus, we suggest that the mechanism of improving effect of nano-curcumin on FPG, insulin, and HOMA-IR might be associated with the decrease in the concentration of serum afamin. Therefore, the nano-curcumin administration could be a preferred choice to improve the treatment of MS and these data may contribute to the management and treatment strategies of MS patients. Therefore, future research needs to investigate the effects of longer treatment periods and other doses of nano-curcumin therapy.
